# Shake It Off: Investigating the Function of a Domestic Dog Behavior in Social Contexts

**DOI:** 10.3390/ani14223248

**Published:** 2024-11-13

**Authors:** Ani Bryce, Paige Nurkin, Alexandra Horowitz

**Affiliations:** Dog Cognition Lab, Department of Psychology, Barnard College, New York, NY 10027, USA; paigenurkin72@gmail.com (P.N.); ahorowit@barnard.edu (A.H.)

**Keywords:** domestic dogs, canine behavior, shaking, behavioral observation, transition, affect

## Abstract

Shaking in domestic dogs—a rapid side-to-side movement of the body or head—is a commonly observed behavior, yet its function remains minimally researched. The present study aimed to investigate the use of shaking behavior in naturalistic social contexts, with the hypothesis that shaking functions as a marker of transition between behaviors or activities. In addition, as the prior literature has suggested that shaking more frequently occurred in postures or conditions of stress, either to signal stress or to mitigate it, we looked at postures related to affect before and after shaking. We observed 96 New York City dogs in two contexts: a public dog run and a private daycare. Over 120 episodes, shaking behavior was most often observed between two different activities or behavioral categories, supporting the hypothesis that shaking functions as a marker of transition between behaviors and activities. We also found neither a significant change in postures related to affect nor in the number of dogs in the subject’s vicinity after shaking. While there may be other functions of canine shaking behavior, this observational research adds to our understanding of a common and understudied dog behavior.

## Simple Summary

While shaking behavior—a rapid side-to-side movement of the body or head—is often seen in domestic dogs, its purpose is not well understood. In this study, we investigated whether shaking acts as a marker of transition between activities or postures associated with affective states. We recorded a total of 120 shakes, from 96 dogs, in a public dog run and a private daycare in New York City, and looked at the behaviors preceding and following the behavior. Shaking most often occurred between two distinct activities or two distinct categories of behavior, supporting the hypothesis that shaking marks a moment of behavioral transition. We did not find that shaking behavior was linked to changes in postures related to affect. This research contributes to our understanding of ubiquitous but understudied behaviors of dogs.

## 1. Introduction

Despite considerable growth over the last two decades in the research on domestic dog (*Canis familiaris*) behavior and cognition [1,2], many foundational behaviors of dogs have not been studied. The majority of recently published papers investigate social cognition in dogs, especially in interaction with humans; much fewer papers have examined the function or meaning of behaviors used intraspecifically. Without a true dictionary of dog behaviors, the field is at risk of misinterpreting or missing behaviors important to the species in interaction with conspecifics or with humans. Indeed, the analysis of dog behavior is permeated by anthropomorphic claims, which are often not supported by empirical research [3]. 

The basic foundation necessary to begin understanding the function of a behavior is detailed observation [4]. An ethological approach, describing patterns of behavior, allows researchers to identify what is actually occurring within a behavior chain, as well as determining the context surrounding it [5]. Several exemplary ethological studies have aimed to identify the context, usage, and function of basic-level dog behaviors. For instance, observational studies of the context of tail wagging, across multiple species, and in dogs specifically, have identified the function and use of this behavior [6,7,8,9]. Studying “gifted word learning” dogs, Sommese et al. (2022) found that head tilting occurred most often when listening to an owner request a toy familiar to them [10]. Research into the dog’s relaxed open-mouth display, used in dyadic play, has identified its function in attracting other dogs’ attention and mediating play behavior [11,12,13]. Examination of the context of a dog’s familiar “guilty look” in an experimental setting revealed its function as a submissive behavior, responsive to a person, rather than a reaction to their own “misbehavior” [12,14]. 

One behavior regularly observed, occurring across settings and breeds, is shaking (sometimes called “shake off” or “wet dog shake”) behavior. While it is used in canid ethograms [15,16,17,18], often classified as a stress behavior, we could find no prior empirical study establishing the context or function of dog shaking behavior. Following prior work, we define “shaking” as a rapid side-to-side movement of the body or head; it has also been described as a “rotation of the body, starting at the head and moving caudally” [16,17]. The behavior is familiarly seen when a dog has gotten wet; however, we are concerned with the behavior when it appears without this cause. (Note that shivering or trembling is also not the shaking herein described.) Previous studies that have clearly described this type of shaking behavior in dogs have mentioned it as a secondary finding when investigating canine stress. Researchers presenting subjects with a range of conditions—including being separated from and reunited with owners [17,18], performing in therapy sessions and agility competitions [16,19], and experimentally induced physical and spatial restriction [15,20] (this research may be considered problematic, given welfare validity concerns [21]) noted instances of shaking directly after the introduction of the experimental condition. Based on this pattern, shaking was hypothesized to manage stress and tension [18,19]. These interpretations align with the popular notion that individuals faced with stressful situations or sensory discomfort try to “shake it off” via this rotational behavior. Anecdotally, however, we have observed shaking in many different contexts, such as when rising, or upon exiting a room or stepping outdoors, which suggests that the behavior may serve to mark a change in activity as well. Other mentions of shaking include studies that featured human interference (such as petting or restraint) as a stimulus, in which the seen shaking behavior was hypothesized to enable dogs to rearrange their fur—as after rising or physical contact [15,17]. Separately, shaking of the head from side to side has been identified as part of social play in canids [4]. In studies of non-canids, shaking behavior has been identified in observational and experimental studies as having a displacement function, or as a response to irritation. In greylag geese, shaking was seen most often after a period of conflict [22]; in Java and long-tail macaques, shaking has been considered a displacement activity, like scratching, yawning, and self-grooming [23,24]. In horses, head shaking may be seen as a behavioral disorder [25].

In the current study, we aimed to identify the behaviors before and after shaking occurs in dogs when in social contexts. We performed ad lib observations of owned dogs in New York City in dog run and dog daycare settings, videotaping and coding the preceding and succeeding behaviors of each shaking episode seen. Given the disparate social and environmental contexts in which shaking has been seen, we hypothesized that shaking behavior would be most often seen at points of transition between activities. Secondarily, we hypothesized that shaking would be seen as a point of transition between postures associated with affective states, and thus may function as a marker of such transition.

## 2. Materials and Methods

### 2.1. Subjects

Shaking behavior was observed in 96 domestic dogs. Demographic data about our subjects, such as age and sex, were limited due to the observational nature of our study. To distinguish dogs, we gathered information on their size (extra small: toy; small: corgi or beagle; medium: border collie or Cocker spaniel; large: Labrador or German shepherd; extra-large: great Dane) and coat length. (No extra small or extra-large dogs were seen.) In total, 34 were small in size, 19 were medium, and 43 were large; 30 dogs had short-length coats, 37 had medium-length coats, and 29 had long-length coats. 

### 2.2. Study Sites

Subjects were observed either in-person at the 105th Street Dog Run in Riverside Park, a public dog run on the Upper West Side of New York City, or virtually at multiple locations of “D is for Doggy”, a private dog daycare service in New York City. Each location of the daycare facility has a remote monitoring system, which continually outputs a video that can be accessed online in real-time during working hours. 

**Figure 1:**
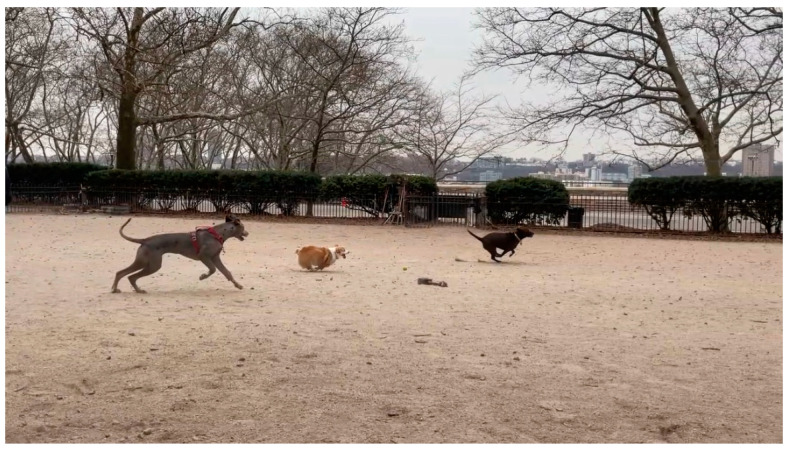
Screenshot of video from observation session at 105th Street Dog Run.

The 105th Street Dog Run is an outdoor, fenced run that is circular in shape and surrounded by trees, bushes, and walking paths (Figure 1). The dog run uses sand and gravel substrate with mulch on the periphery. There are four benches along the inside perimeter for owners, a water fountain to wash dogs, and a box of provided tennis balls. The dog run requires that all dogs are vaccinated, and that owners must always remain with their dog. The number of dogs in the run at one time during observational sessions ranged from 3 to 16, and the number of owners typically matched the total dogs with the occasional additional person per dog, or additional dog per person. The number of dogs in the run fluctuated every few minutes as dogs and owners arrived and departed. Dogs differed in the amount of time that they interacted with people or other dogs, or spent alone.

**Figure 2:**
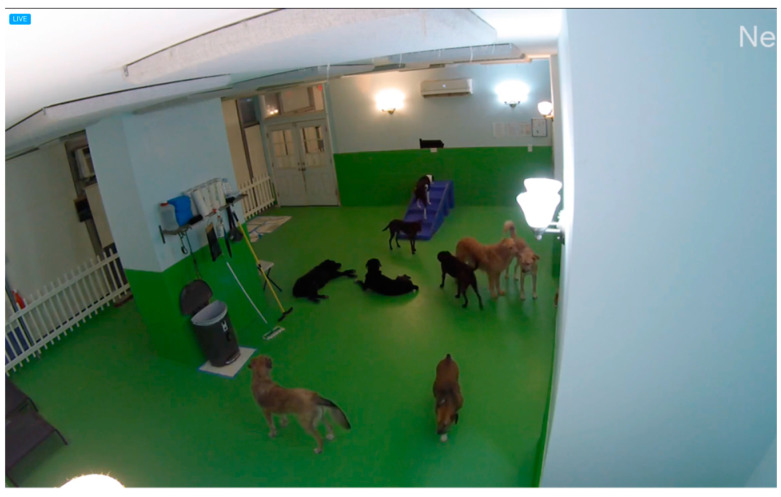
Screenshot of video from observation session of the D is for Doggy live stream at the Amsterdam Avenue location (Room 1).

The “D is for Doggy” daycare locations are all indoors, and range from single rooms to connected double rooms, all with green epoxy flooring (Figure 2). Some locations have windows to the street outside, while others have no windows. All locations have a plastic staircase structure with a tunnel cut out on the underside for dogs to run through, as well as at least one elevated cot made of trampoline material. Visible at each location are also several pads for the dogs to urinate or defecate on, and cleaning materials. At some locations, bowls of water were visible. The number of dogs in the daycare at one time depended on the location but ranged from 8 to 34. Each location always had between one and three daycare workers visible. The number of dogs in the daycare remained consistent throughout an observational session except during evening sessions, when subject numbers dropped as owners retrieved their dogs. Each dog in the daycare varied in their level of engagement with the other dogs and with the daycare workers.

### 2.3. Behavioral Data Collection

Nineteen hours of observational data were collected, from February to March 2023. Observations were performed at least twice per week for an hour per session, and observers alternated between the dog run (in-person) and daycare (virtual) settings, resulting in a total of ten recorded observation sessions of the dog run and nine recorded observation sessions of the daycare. In the dog run setting, observers used iPad cameras to film the entire area of the run for the duration of the session. The daycare footage was publicly available online. For the daycare setting, observers used the computer screen recording feature to film the entire webcam screen for the duration of the session. At the start of each observational session, we recorded location conditions (weather, substrate, owners’ and dogs’ proximity, and time). In the dog run setting, observers entered the run without interacting with the dogs to reduce interference in the natural behavior of the dogs in the run, and they filmed from a bench. Observers did not interact with owners unless owners asked why they were filming or what they were doing in the dog run without a dog. When requested, an informational sheet about the study was provided.

### 2.4. Behavioral Coding 

All observation sessions were coded at a later date from video recordings; summary notes were taken at the time of observation to aid later coding. When coders identified a “shake”, the shaking dog became the focal subject. A shake was characterized as either “full”, for a full-body shake, or “head only”, if only the head was shaken. For each focal subject, all behaviors of the subject were coded from ten seconds pre-shake to ten seconds post-shake [12,26]. Behaviors were drawn from an ethogram (Table 1) compiled from previous research, and they were intended to capture the physical and social behaviors present in a social setting. For each behavior, its presence (1) or absence (0) in the twenty-second period was indicated on a data sheet. If multiple behaviors were observed within one ten-second period, a primary behavior or behaviors and any secondary behaviors were noted. The determination of what behavior was primary was made by considering what the dog was primarily doing in the entire ten-second period; secondary behaviors were ancillary behaviors on the ethogram that also occurred in that time. However, all behaviors observed in the sample period were recorded on the ethogram and were included in our analysis. We additionally grouped the individual behaviors into seven distinct behavioral categories according to their similarity: play; charged social interactions; casual social interactions; person interactions; grooming; by oneself; and entering/exiting the location.

We also coded indicators of a subject’s physical and social state to speak to, as far as possible from behavior, a subject’s affect at the same time points pre- and post-shake, including body posture, ear position, tail position, tail wag, the number of dogs within a three-foot (roughly 1 m) radius, and the number of humans within a three-foot radius [27,28]. Postural states were coded on the ethogram based on a numerical range: for ear and tail, either low (0), middle (1), or high (2) positions; for body posture, supine (0), prone (1), sit (2), stand (low or very low) (3), or stand (high or neutral) (4). These postural states, while not precise measures of a dog’s affect, give some gauge of the dog’s arousal level, so we would expect them to change (as, e.g., from a low tail to a high tail) if the arousal level of the dog changed. We coded the number of other proximate (within 3 feet/1 m) dogs or people in order to see if shaking served to prompt others to leave the subject’s proximity. If a subject was not visible on camera either pre or post shake, behaviors were not coded and were marked as out of view (OOV).

### 2.5. Data Analysis

Video recordings of subject behaviors were later viewed in frame-by-frame playback and coded per the ethogram. In order to interpret our ethogram data, we summed occurrences of each behavior included in the ethogram pre-shake and post-shake. We also tallied the number of instances of each behavioral category both pre-shake and post-shake. Data for both the individual behaviors and the behavioral categories were used to run statistical analyses. We also analyzed changes in body posture of the dogs pre- and post-shake and observed the number of dogs near a subject before and after a shaking incident occurred. 

After summing data points, we used chi-square tests of independence and transition matrices to compare behaviors pre- and post-shaking—behavioral transition—and chi-square tests of independence to compare both postures and proximity pre- and post-shaking. Behavioral coding reliability was determined by comparing the identified behavior of the main coder (AH) against a second coder (PN). Across all recorded sessions, coders had complete agreement in identifying shaking behaviors, either full or head-only (*n* = 120; Cohen’s Kappa = 1.0). To gauge inter-observer agreement for behaviors occurring pre- or post-shake, we created a contingency table for inclusion or exclusion of behaviors at each time point. When adjusted to exclude correlations due to chance, reliability was high (*n* = 40; Cohen’s Kappa = 0.82).

## 3. Results

In total, we observed and coded 120 shaking episodes (100 full-body; 20 head-only) from 96 unique dogs across 19 observation periods.

### 3.1. Behavioral Transition

In order to investigate whether shaking marked a transition in behavior, we compared behaviors pre- and post-shaking. In 107 of 120 shaking instances, different behaviors were observed at each timepoint. Chi-square tests indicated that shaking marked a moment of behavioral transition [χ^2^ (1, *n* = 120) = 73.63, *p* = 0.01]. Similarly, when behaviors were grouped into categories, there was a complete (all behavior groups differed pre- and post-shake) or partial (at least one behavior group differed pre- and post-shake) change in 100 of the 120 observed instances. A chi-square test indicated that shaking marked a change in behavioral category significantly more than expected by chance [χ^2^ (1, *n* = 120) = 17.45, *p* = 0.01]. Both of these findings support the hypothesis that shaking occurs at points of behavioral transition. 

To further investigate behavioral transitions, we created transition matrices to visualize proportions of behaviors before and after shaking (Table 2 and Table 3). (Note that we could not conduct a Markov analysis with the matrices, since the data sheet allowed for more than one pre-shake or post-shake behavior, and, thus, each row in the matrix did not add up to a probability of one.) Table 2 shows the six most frequently observed behaviors, while Table 3 is of the seven behavioral categories. In a few cases, the matrices show some continuation of a behavior after a shake: specifically, when play (as play with dogs or the behavioral category play) was a pre-shake behavior, it was also a post-shake behavior over half of the time. This is also true for the behavioral category by oneself. However, for all ten other behaviors or behavior categories (still, walk alone, walk toward other dogs, move toward person, sniff other dog, charged social interaction, person interaction, casual social interaction, grooming, and enter/exit), there was a change in behavior or behavior category from pre- to post-shake most of the time. This supports the idea that shaking is most often a transitional behavior.

To ensure that shaking behavior was not simply a response to something on their body, we looked at the proportion of shakes that occurred after physical contact with an external object (such as the ground, another dog, or a person touching). Most shaking episodes (79/120) were not a result of external contact [χ^2^ (1, *n* = 120) = 12.03, *p* = 0.01]. 

### 3.2. Changes in Posture and Social Proximity

We looked at whether there were various postural changes from pre- to post-shake. Tail height was more often the same before and after shaking [χ^2^ (1, *n* = 114) = 58.98, *p* < 0.0001], as was ear height [χ^2^ (1, *n* = 119) = 69.59, *p* < 0.0001] and body position [χ^2^ (1, *n* = 120) = 61.63, *p* < 0.0001]. The presence or absence of tail wag was also more often the same before and after shaking [χ^2^ (1, *n* = 114) = 15.97, *p* = 0.0001]. These results suggest that the shake was not serving to change the dog’s body posture, and, insofar as the body posture is related to arousal or affect, the dog’s arousal level or affect. With regard to environmental context—specifically, the number of dogs in the vicinity—we also looked at whether the shake effected a change. The null hypothesis, that shaking did not affect a decrease in the number of dogs in the focal dog’s vicinity, was supported [χ^2^ (1, *n* = 120) = 1.2, *p* = 0.01]. In only 28% of cases (34 of 120 shakes) did the number of dogs decrease after the shake; 72% of the time, the number of dogs stayed the same or actually increased.

## 4. Discussion

We performed a naturalistic observational study using continuous recording, which allowed later focal sampling of individual animals’ behavior. We observed that in social contexts, shaking behavior in dogs most often bridged a change from one behavior to another behavior, or a transition from one behavioral category to another behavioral category. This finding supports the hypothesis that shaking is a marker of behavioral transition. 

In some respects, this suggests that shaking may serve an analogous function to “cut-off” behaviors [29], which were suggested to be used to disengage from an activity. As Chance described them, these acts were used in agonistic contexts [29]; shaking behavior, by contrast, is very often used in affiliative and non-agonistic contexts. Still, insofar as they both serve to change the current behavior, they may be similar.

Shaking has been widely asserted to be an indicator of stress. We did not directly test this hypothesis; however, some of the measures that we were able to record observationally do not entirely support these assertions in these social contexts. For instance, looking at postures associated with affective states, including tail wag, tail position, body posture, and ear position, only a minority of the time did the posture change after the shake. We also found that the number of dogs near the subject after shaking did not significantly increase or decrease, which suggests that shaking was not a deterrent to other dogs, nor a signal indicating to other dogs to avoid them. In fact, in several (35) cases, a shake seemed to attract dogs, insofar as it led to more dogs in the shaking dog’s vicinity. Again, we were not measuring stress directly, only looking at behavioral parameters widely understood to indicate more negative or positive affect. Thus, this result should not lead to a conclusion that shaking is not in any way related to or exhibited because of stress, but it does suggest that shaking behavior is very often used in ways unrelated to an experience of stress.

This unexpected finding contradicts those aforementioned studies that marked shaking as solely a stress behavior. One explanation for this divergence is that that those studies saw shaking observed after either an experimentally introduced stimulus such as physical restraint, a surprising object (e.g., umbrella), separation from owners, being left with strangers, or a structured event such as a therapy dog session or agility contest [15,16,17,18,19,20]. As each of these studies had a primary aim of investigating dog stress responses, it is not surprising that in a presumably stressful circumstance, any observed shaking was thought to be related to stress. In contrast, we were looking at naturalistic intraspecific social contexts, which may more accurately reflect dogs’ ordinary use of the behavior. Further research may attempt to gather physiological measures of stress, not just behavioral ones, such as cortisol levels and heart rate, which could add to this interpretation. 

Future research may attempt to characterize all behaviors, not only those around the focal animal’s shake. As we did not look at focal dog behavior outside of the twenty seconds pre- and post- shaking, we could not compare the pre- and post-shake behaviors to the overall patterns of behavior of the subject group. Such a study would give more information about whether shaking serves as a unique role in behavioral transitions, and whether certain behaviors are more likely to be preceded or followed by shaking. Relatedly, by coding only ten seconds pre- and post-shake, we may have missed behaviors that were relevant to the shake; however, we believe that a survey of the most proximate behaviors is a reasonable and useful place to start. Further, by characterizing multiple behaviors that were occurring in our sample period, we limited the power of the transition matrices to show patterns in behavior. That said, it would have been inappropriate to limit the description of the subjects’ behavior to just one behavior. In fact, the matrix patterns seen mostly supported the finding that shaking mediated behavioral transitions. In the primary case where it did not—around play behavior—it is possible that shaking served as an attention getter to restore a paused play session [12].

Notably, because we examined shaking behavior in a social context, any conclusions may be limited to the use of shaking behavior in social contexts, and may not extend to shaking behavior in other contexts. And in particular, our observations were of a dog run and dog daycare, and behaviors in those contexts may not generalize to all other contexts, such as unrestricted social space. We did not compare shaking behavior between dog run and daycare contexts, though this may be informative, as daycares may be much smaller spaces with many more dogs. A denser social environment could influence the focal subject and result in different rates of behaviors. The observational nature of our study restricted our ability to gather physiological information that may be relevant to further interpretation of the behavior of shaking. Nonetheless, observational work also increases opportunities to see natural species behavior, rather than behavior in a limited experimental setting.

## 5. Conclusions

By observing intraspecific interactions, we have found that shaking serves to mark behavioral transitions. This identified function does not preclude other functions. Notably, though, it differs from its use in dog ethograms, which use was not founded on observational work. It is critical for the science that we do not misclassify or prematurely label commonly observed behaviors, nor should we assume that one behavior has only one meaning. Basic dog behaviors should continue to be empirically investigated; our assumptions about their use and meaning may be premature and oversimplified.

## Figures and Tables

**Table 1:** Ethogram used to analyze the behavior and position of dogs.

**Behavior**	**Description**	**Behavioral Category**
Play	Excited, reciprocated interactions, with mutual participation; note if with dog or human	play
Play bow	Forelimbs down; hind end raised; tail erect or wagging	
Play alone	Play with object	
Subject chase other dog	Any chasing behavior involving the subject running after another dog	
Other dog chase subject	Any chasing behavior involving another dog running after the subject	
Chase object	Running directly toward object	
Fight	Aggressive behavior—including pinning/wrestling, and mouthing, without play markers— displayed by one or multiple dogs	charged social interaction
Non-reciprocated play	Display of play signals or behavior, not engaged by other dog who may be showing signs of stress; note who is focal dog and who is other dog	
Run towards/from other dogs	Movement at fast trot or faster toward or away from other dogs	
Hump	Mount front or rear of dog with pelvic thrusting; note focal or other dog	
Bark	Harsh, noisy vocalization in proximity to and directed toward other dog	
Other dogs play/scuffle (<3 ft)	Dogs engaged in play near focal dog	
Walk towards/from other dogs	Movement at a regular pace where at least two feet are on the ground towards or away from other dogs	casual social interaction
Subject sniffs other dog	Subject brings nose to other dog	
Other dog sniffs subject	Other dog brings nose to subject	
Other dog full/partial/head shake (<3 ft)	Other dog engages in rapid side-to-side movement of the body or head	
Run from human	Movement at fast trot or faster away from human	person interaction
Walk from human	Movement at a regular pace where at least two feet are on the ground away from human	
Person touch	Human touches body of dog on purpose; note if positive (petting) or negative (restraint)	
Person approach	Human moves directly toward subject	
Person call dog	Human cues subject vocally to get attention	
Moves towards person	Subject directly approaches human	
Chase person	Any chasing behavior involving the subject running after a human	
Person chases dog	Any chasing behavior involving a human running after the subject	
Sniffs person	Subject brings nose to human	
Looks at person	Subject fixates eyes on human	
Take off leash	Human removes leash from dog	
Take off harness/collar	Human removes harness or collar from dog	
Put on leash	Human attaches leash to dog	
Put on harness/collar	Human applies harness or collar to dog	
Subject sniffs self	Subject brings nose to its own body	grooming
Urinating	Lifting a hind leg or squatting to excrete urine	
Defecating	Hunched, squatting position to discharge excrement	
Drinking water	Uses tongue to lap up water	
Stretching	Subject extends body and legs away from each other	
Still	Absence of subject movement	by oneself
Scratch self	Subject repeatedly rubbing own body quickly with a paw(s)	
Run alone	Movement at fast trot or faster separate from other dogs or humans	
Walk alone	Movement at a regular pace where at least two feet are on the ground	
Subject sniffs ground	Subject brings nose to ground	
Subject sniffs air	Subject lifts nose into air	
Yawn	Opens mouth and stretches it open widely	
Paw at ground	Uses paws to scrape or dig at the ground	
Rolling on ground	Lies down and rotates body side to side on ground	
Body contact with object	Body of subject touches an inanimate object in environment	
Entering location	Subject moves through entryway of location to occupy location	entering/ exiting
Exiting location	Subject moves through entryway of location to depart location	
**Position**	**Description**	**Position Category**
Tail position	Low: tail tucked between the legs or positioned significantly lower than the rump and closer to the legs Neutral: tail positioned farther from the legs and relaxed High: tail as high as can be held	body position
Tail wag	Presence of lateral tail movement	
Body posture	Stand high: tail up, head up, body erect, all legs upright + on ground Stand neutral: tail low, ears back, 2 or more legs bent + on the ground Sit: rump on the ground, front legs straight, back legs bent, body erect Lie (prone): lie on stomach or side with face down Lie (supine): lie on back or side with face up	
Ear position	Back: folded against head Neutral: relaxed Alert: perked or held forward	
Piloerection	Hair raised (“hackles”) behind shoulders or on back/rump	
Number of people nearby	Within a 3-foot radius of focal dog	social position
Number of dogs nearby	Within a 3-foot radius of focal dog	

**Table 2:** Transition matrix of the sequences of the six most frequently occurring behaviors. Each cell indicates the proportion of row behaviors (pre-shake) that were followed by the column behavior (post-shake), with respect to all six behaviors.

Pre-Shake Behavior	Post-Shake Behavior					
	Play with dogs	Still	Walk alone	Walk towards other dog(s)	Move towards person	Subject sniffs other dog
Play with dogs (32)	0.531	0.1875	0.0625	0.125	0.1875	0.125
Still (20)	0.05	0.3	0.2	0.2	0.25	0.2
Walk alone (19)	0.053	0.368	0.421	0.182	0.083	0.154
Walk towards other dog(s) (11)	0.182	0.364	0.091	0.182	0.091	0.091
Move towards person (12)	0.25	0.167	0.333	0.167	0.083	0
Subject sniffs other dog (13)	0	0.538	0.077	0.077	0.154	0.154

**Table 3:** Transition matrix of the seven behavioral categories. Each cell indicates the proportion of row categories (pre-shake) that were followed by column categories (post-shake), with respect to all seven behavioral categories.

Pre-Shake Behavior	Post-Shake Behavior						
	Play	Charged social	By oneself	Person interaction	Casual social	Grooming	Enter/Exit
Play (38)	0.553	0.158	0.184	0.211	0.289	0	0.026
Charged social (22)	0.273	0.318	0.273	0.364	0.409	0	0.045
By oneself (44)	0.25	0.068	0.523	0.091	0.318	0	0.023
Person interaction (34)	0.029	0.147	0.382	0.441	0.353	0	0.029
Casual social (27)	0.222	0.185	0.481	0.148	0.407	0	0
Grooming (5)	0	0	0.6	0.2	0.4	0	0
Enter/Exit (1)	1	0	0	0	0	0	0

## Data Availability

The data presented in this study are available upon request from the corresponding author.
